# When to Use Rectangular Waveforms in Dielectrophoresis Application to Increase Separation and Sorting Efficiency

**DOI:** 10.1002/elps.202400164

**Published:** 2024-11-28

**Authors:** Niklas P. Boldt, Laura Weirauch, Jana M. Späth, Uwe Kerst, Mario Birkholz, Michael Baune, Roland Thewes

**Affiliations:** ^1^ Chair of Sensor and Actuator Systems Faculty of EECS, TU Berlin Berlin Germany; ^2^ Faculty of Production Engineering Chemical Process Engineering University of Bremen Bremen Germany; ^3^ Integrated Electronic Systems Lab (IES) Technical University of Darmstadt, Darmstadt Germany; ^4^ IHP ‐ Leibniz‐Institut für innovative Mikroelektronik Frankfurt (Oder) Germany

**Keywords:** cross‐over frequency, dielectrophoresis, harmonics, microfluidic, microparticle, waveform

## Abstract

In this study, the influence of using rectangular waveforms is comprehensively investigated on the separation and sorting efficiency of dielectrophoretic (DEP) processes. Besides positive effects on DEP experiments, cases of a diminished force due to rectangular waveforms are investigated and discussed. This investigation encompasses two primary experimental setups. First, microparticle‐focusing experiments are carried out using a pair of electrodes within a microfluidic channel. Second, separation experiments are performed using a macroscopic insulator‐based dielectrophoretic filter. The study reveals that harmonics of rectangular signals can have a positive impact on separation or sorting efficiency when compared to sinusoidal waveforms, provided that these harmonics contribute to the overall DEP force with the same sign. This positive effect is found to depend on the ratio between the applied fundamental frequency and the cross‐over frequency in the Clausius–Mossotti factor. However, violating related derived boundary conditions leads to negative effects and a decrease in the DEP net force.

**Abbreviations**
CM: Clausius–Mossotti
DEP: dielectrophoresis
DI: deionized
nDEP: negative dielectrophoresis
pDEP: positive dielectrophoresis
pp: peak‐to‐peak
PS: polystyrene
rms: root‐mean‐square

## 1 Introduction

Dielectrophoresis (DEP) is a widely used technique for manipulating microscale particles within heterogeneous mixtures in both biological and chemical applications. Typically, sinusoidal waveforms are employed due to their focused signal energy at a single frequency, which helps to avoid cross effects with other frequencies [1].

Considering a simple spherical particle or cell, the strength of the dielectric force is primarily determined by the electrical field strength and the radius of the particle, as described by the following equation:

(1) $${\vec{F}}_{\text{DEP}}=2\pi R_{p}^{3}\varepsilon_{m}\text{RE}\left\{\text{CM}\left(\omega \right)\right\}\nabla {\left|{\vec{E}}_{\text{rms}}\right|}^{2},$$

where $R_{p}$ is the radius of the particle, $\varepsilon_{m}$ is the permittivity of the medium, $E_{\text{rms}}$ is the root mean square of the electrical field applied to the electrodes, and CM is the frequency‐dependent Clausius–Mossotti factor.

With the complex permittivity $\varepsilon_{i}^{*}=\varepsilon_{i}\varepsilon_{0}-j\frac{\sigma_{i}}{\omega }$ (where i=p,m for particle and medium), the CM factor is given by

(2) $$\text{CM}\left(\omega \right)=\frac{\varepsilon_{p}^{*}-\varepsilon_{m}^{*}}{\varepsilon_{p}^{*}+2\varepsilon_{m}^{*}}.$$

The real part of the CM factor amounts to [2]

(3) $$\text{RE}\left\{\text{CM}\left(\omega \right)\right\}=\frac{\omega^{2}\left(\varepsilon_{p}-\varepsilon_{m}\right)\left(\varepsilon_{p}+2\varepsilon_{m}\right)+\left(\sigma_{p}-\sigma_{m}\right)\left(\sigma_{p}+2\sigma_{m}\right)}{\omega^{2}{\left(\varepsilon_{p}+2\varepsilon_{m}\right)}^{2}+{\left(\sigma_{p}+2\sigma_{m}\right)}^{2}}.$$

The frequency dependence of RE{CM(ω)} with the permittivity $\varepsilon_{p}$, $\varepsilon_{m}$ and the conductivity $\sigma_{p}$, $\sigma_{m}$ of particle and media can result in a change of sign. That leads to a DEP force, which acts repulsively or attractively towards regions of high electrical field gradients. This phenomenon is also referred to as negative or positive DEP (nDEP or pDEP), respectively. This can then be used, for example, to separate cells of different types, such as blood cells [3], or cells with different properties, such as microalgae with different content of fatty acids [4].

With ω=2πf, it is evident that the force directly depends on the applied frequency. Since an ideal sinusoidal signal spectrally provides a single frequency, its widespread use in DEP applications is well justified.

In some applications, the use of rectangular waveforms and their higher root‐mean‐square (RMS) amplitude—which spectrally consists of multiple frequencies—are advantageous in terms of the efficiency of trapping particles with pDEP [5, 6] or in a better alignment of multiwall carbon nanotubes [7]. Such waveforms enhance the effect and efficiency in sorting or trapping of the DEP application in specific scenarios. This can be particularly advantageous for DEP processes in which particles are focused on a streamline in order to be separated or sorted. Examples include continuous processes such as particle deflection into different outlets using (three‐dimensional) electrodes [8, 9] or insulating structures [10, 11]. Processes in which particles are retained or trapped via DEP, such as in DEP filtration processes [12, 13, 14], can also benefit from a simple approach to increase the DEP force. However, the use of rectangular waveforms is not always beneficial and can lead to worsening of the DEP force.

This work addresses the use of rectangular waveforms in DEP experiments, gives a deeper theoretical understanding of the spectral nature of those signals, and provides a guideline under which circumstances the use of rectangular signals is beneficial. Moreover, this work points out cases where the use of rectangular signals is disadvantageous leading to a decrease in total DEP net force.

## 2 Theoretical Background

In most lab‐based DEP applications, commercially available signal generators are used to provide standard waveforms such as sine, rectangular, ramp, triangle, and others. By the use of such devices, the electrical field strength at DEP electrode structures is limited by the maximum amplitude of the device. This relates to a constraint for induced energy and DEP force in the used setup.

Considering the difference between a rectangular and sinusoidal waveform, the rectangular waveform is a superposition of an infinite number of sine waves with increasing frequency and decreasing amplitude. This is defined by the Fourier series expansion:

(4) $$⊓\left(t\right)=\frac{4}{\pi }V_{0}\sum_{k=1}^{\infty }\frac{\sin(2\pi (2k-1)ft)}{2k-1}.$$

**FIGURE 1:**
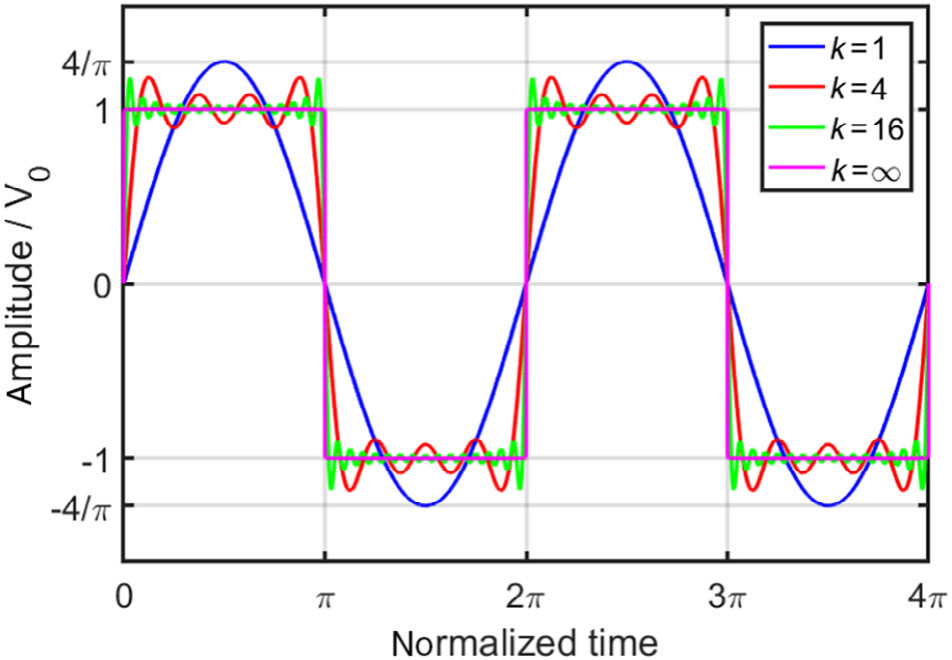
Rectangular waveform as a Fourier series expansion with different expansion indices k of a sinusoidal waveform with amplitude $\frac{4}{\pi }V_{0}$.

Therefore, a rectangular waveform with frequency $\omega_{f}$ and amplitude $V_{0}$ can be built out of an infinite series of sine waves with a fundamental frequency of $\omega_{f}$, but an amplitude of $\frac{4}{\pi }V_{0}$ in the time domain as depicted in Figure 1.

**FIGURE 2:**
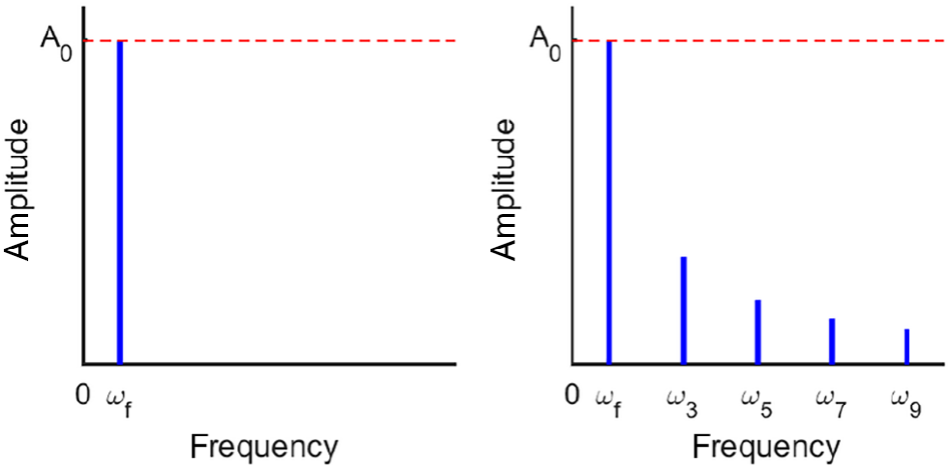
Comparison of the spectral response of a sine and a rectangular waveform signal. The sine wave (left) has only one peak at the fundamental frequency $\omega_{f}$, whereas the rectangular waveform (right) has the same peak at $\omega_{f}$ but an infinite number of odd harmonic distortions with subsiding amplitude with $\frac{4}{\pi n}$.

For the spectral domain, the multifrequency nature of the rectangular waveform becomes more distinct in comparison to the time domain as depicted in Figure 2. The amplitude of fundamental frequency $\omega_{f}$ is equal for both sine and rectangular waveforms, but the rectangular waveform provides additional frequencies at $\left(2n-1\right)\omega_{f}$ with decreasing amplitude. Since each amplitude in the spectral domain has a certain energy, it can be concluded that the total energy of a rectangular waveform must be larger, since it has additional frequencies compared to a perfect sinusoidal waveform.

In [5, 6, 7], the effective amplitude is taken into account to argue for a higher energy of the rectangular waveform. The effective amplitude of an AC signal determines the DC amplitude equivalent which dissipates the same power in an ohmic load and therefore is directly related to the energy of a signal. It is also referred to as the root‐mean‐square (rms) amplitude which is given for both signals by

(5) $$V_{\text{rms,sine}}=\frac{1}{\sqrt{2}}V_{0}$$

for a sinusoidal waveform and

(6) $$V_{\text{rms,rect}}=V_{0}$$

for a rectangular waveform. Moreover, the effective amplitude relates to the same peak amplitudes of sinusoidal and rectangular waveforms, unlike the Fourier expansion of Equation (4). For a rectangular and sinusoidal waveform with the same peak amplitude $V_{0}=1$ in the time domain, the spectral amplitude of the corresponding fundamental frequency for the rectangular waveform increases to $\frac{4}{\pi }$, along with corresponding harmonic frequencies.

From the definition of signal energy

(7) $$P=\int_{-T/2}^{T/2}V\left(t\right)dt,$$

it is straightforward to calculate that a rectangular waveform has twice the energy of a sine waveform with the same peak amplitudes. This holds true in both the time and frequency domain due to Parseval's theorem [15]. However, the energy is distributed over multiple frequencies and not focused on a single frequency like which is the case for a sinusoidal waveform. This difference in energy distribution requires a closer consideration in using rectangular waveforms, due to the frequency dependence of the DEP force.

Considering Equation (1) and the multifrequency nature of a rectangular waveform, the total DEP force can be described as a summation of the force at the fundamental frequency and the forces contributed by the harmonic frequencies [16]. This relationship can be expressed as follows:

(8) $${\vec{F}}_{\text{DEP,tot}}={\vec{F}}_{\text{DEP}}\left(\omega_{0}\right)+\sum_{i=1}^{N}{\vec{F}}_{\text{DEP}}\left(\omega_{i}\right)$$

(9) F⃗DEP,tot=F⃗DEP,fundamental+F⃗DEP,harmonics.

Since the total signal energy–and consequently the DEP force–is distributed across multiple frequencies, there is a possibility that the additional forces generated by the harmonics may have an opposite sign compared to the force at the fundamental frequency. This can result in a reduction of the overall net force [16].

Given this scenario, the sum of the forces induced by the harmonics in Equation (10) can be separated into distinct components:

(10) $${\vec{F}}_{\text{DEP,harmonics}}=\underset{A}{\underset{︸}{\sum +{\vec{F}}_{\text{DEP}}}}+\underset{B}{\underset{︸}{\sum -{\vec{F}}_{\text{DEP}}}}.$$

Here, term A represents the sum of all forces which contribute to an increase in the total DEP force, while term B represents the sum of all forces which contribute to a decrease in the total DEP force.

From this, we can conclude that if |A| is bigger than |B|, the overall net force, ${\vec{F}}_{\text{DEP,tot}}$, can be enhanced solely by employing a rectangular waveform.

This condition, where |A|>|B|, can be further elucidated through a mathematical analysis of the rectangular signal and its energy in the spectral domain.

The amplitude of any harmonic of an ideal rectangular waveform is given by

(11) $$A_{n}=\left\{\begin{array}{cc} \frac{4}{\pi n}, & \text{if}\;\text{n is odd}\\ 0, & \text{if}\;\text{n is even.} \end{array}\right.$$

The spectral energy for any harmonic therefore is

(12) $$P_{n}=A_{n}^{2}=\left\{\begin{array}{cc} \frac{16}{\pi^{2}}\frac{1}{n^{2}}, & \text{if}\;\text{n is odd}\\ 0, & \text{if}\;\text{n is even.} \end{array}\right.$$

With this, the total spectral energy for all frequency components of an ideal rectangular waveform with an infinite number of odd harmonics can be described as

(13) $$P_{\text{tot}}=\frac{16}{\pi^{2}}\sum_{n=1}^{\infty }\frac{1}{{\left(2n-1\right)}^{2}}.$$

The sum can also be expressed as

(14) $$\sum_{n=1}^{\infty }\frac{1}{{\left(2n-1\right)}^{2}}=\sum_{n=1}^{\infty }\frac{1}{n^{2}}-\sum_{n=1}^{\infty }\frac{1}{{\left(2n\right)}^{2}}=\frac{3}{4}\sum_{n=1}^{\infty }\frac{1}{n^{2}}.$$

With the known sum of $1/n^{2}$ converging to [17]:

(15) $$\sum_{n=1}^{\infty }\frac{1}{n^{2}}=\frac{\pi^{2}}{6},$$

we can conclude that the sum of all odd harmonics equals:

(16) $$\sum_{n=1}^{\infty }\frac{1}{{\left(2n-1\right)}^{2}}=\frac{3}{4}\frac{\pi^{2}}{6}\approx 1.234,\;$$

(17) $$=\underset{\text{fundamental freq.}}{\underset{︸}{\sum_{n=1}^{1}\frac{1}{{\left(2n-1\right)}^{2}}}}+\underset{\text{harmonics}}{\underset{︸}{\sum_{n=2}^{\infty }\frac{1}{{\left(2n-1\right)}^{2}}}}$$

(18) =1+0.234.

Given that the sum of all harmonics for n>1 is 0.234, the total induced DEP force will be increased if at least 50% or 0.1168 of that energy contributes to the force generated by the fundamental frequency. Calculating the first harmonics and summing them up, we find that

(19) $$\sum_{n=1}^{\infty }\frac{1}{{\left(2n-1\right)}^{2}}\approx 1+\underset{=0.1511}{\underset{︸}{0.1111+0.04}}+\sum_{n=4}^{\infty }\frac{1}{{\left(2n-1\right)}^{2}}.$$

The energy of the third and fifth harmonic is larger than 50% of all harmonic distortions. Therefore, it can be concluded that as long as the sign of the third and fifth harmonic of a rectangular waveform have the same orientation as the fundamental frequency the overall DEP force will increase and the use of a rectangular waveform is beneficial. This effect increases for realistic setups since they are bandwidth‐limited, which leads to an effective decrease of higher order harmonic energies.

In terms of cross‐over frequency $f_{\text{co}}$ of the CM factor and signal frequency $f_{0}$ the condition above reads

(20) $$f_{0}<\frac{f_{\text{co}}}{5}.$$

From this, it can be calculated at which frequency $f_{0}$ the use of rectangular waveforms is advantageous if the cross‐over frequency is higher than the signal frequency. For signal frequencies larger $f_{\text{co}}$, the net force will always increase.

It is important to note that this is primarily valid if the CMfactor of the particle or cell has only one cross‐over frequency, like polystyrene particles. For multishell particles or cells with multiple cross‐over frequencies of the CM factor, it is essential to consider the number of first‐order harmonics which contribute to the sign of the fundamental frequency as estimated in Equation (20).

## 3 Materials and Methods

To evaluate the formerly discussed conditions on an experimental basis, qualitative and quantitative experiments are performed to demonstrate when the use of rectangular waveforms is beneficial or not. For this, an electrode‐based particle focusing experiment is performed and an insulator‐based DEP filter mesh to trap particles is used.

### 3.1 Particle Focusing in an Electrode‐Based Flow Cell Experiment

**FIGURE 3:**
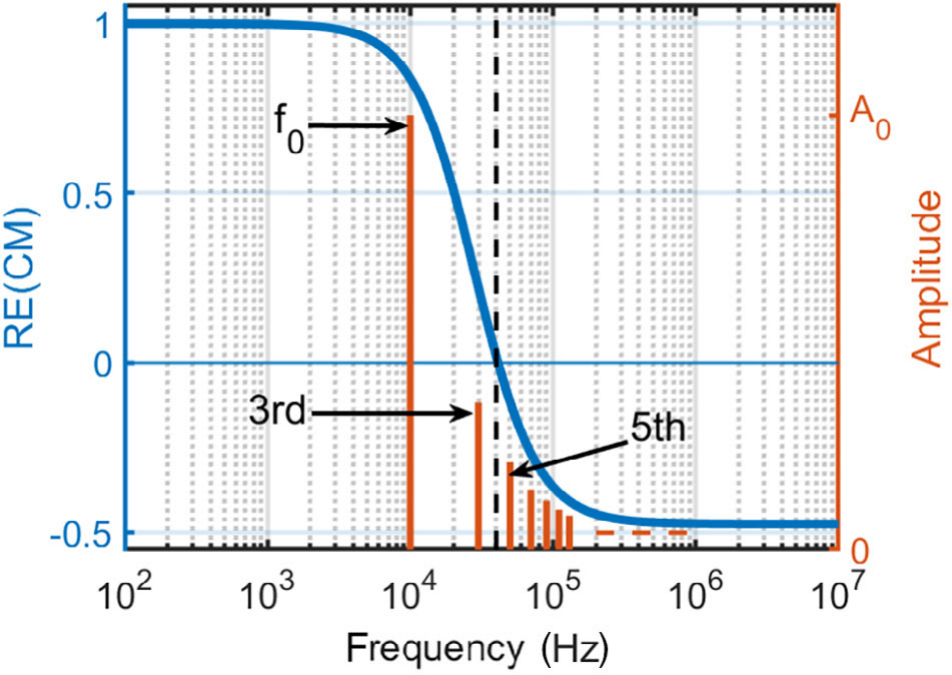
Blue: Real part of the CM factor of 4.5μm carboxyl functionalized polystyrene microspheres [18] in DI water. The approximated zero crossing of RE(CM) is at ≈40kHz. Red: Spectral response of a 10‐kHz rectangular waveform with amplitude $A_{0}$ and the first six harmonics. The fundamental frequency and first harmonic contribute to a positive DEP force. Higher order harmonics will diminish the net force. The real part of the CM factor is calculated by using *myDEP* [20]

For the particle focusing experiment 40μL carboxyl functionalized polystyrene beads [18] of the size $r_{p}=4.5\;\mu m$ are diluted in 1.5 mL deionized (DI) water resulting in a particle concentration of approximately $1.33\times 10^{7}$ particles per milliliter. This solution flows through a microfluidic setup with a top‐bottom electrode structure. The complete setup is described in [16]. Since the impedance of DI water is high, no additional impedance matching is necessary [19] and the flow cell is connected directly to an Agilent 33250A signal generator. For this particle–media combination, the real part of the CM factor is depicted in Figure 3.

Two frequencies of 10 and 350 kHz are used to examine the case that |A|>|B| and |A|<|B|. So for one case, the net force should increase and for the other case the net force should decrease. Both frequencies are compared using rectangular and sinusoidal waveforms and a peak‐to‐peak amplitude of 10 V.

Ten kilohertz is used to be close to the cross‐over frequency so that only the third harmonic will have the same sign as the fundamental frequency as depicted in Figure 3. By this, a pDEP force is induced, and the case |A|<|B| is investigated. For |A|>|B|, 350 kHz is used in inducing an nDEP force, so that all harmonics have the same sign.

**FIGURE 4:**
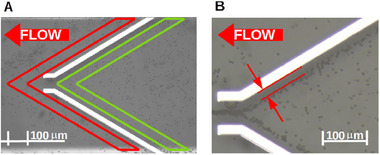
(A) Areas for counting particles for pDEP experiment. Red defines the area where the particles are counted which were not trapped by the pDEP force and green the area where all particles are counted that flow to the electrode structure. (B) Distance between electrode edge and particle stream to evaluate the strength of induced nDEP force.

For a quantitative evaluation of the results, the experimental videos are analyzed in the Fiji‐imageJ software [21]. For pDEP or case |A|<|B|, two areas as depicted in Figure 4A are defined, and the number of particles flowing through this area is evaluated.

The red area counts the particles which could not be trapped between the electrodes. The green area counts all particles which flow towards the electrodes. From those values, a failure rate (FR) in % is defined with

(21) $$\text{FR}=\frac{\text{Total particles in the red area}}{\text{Total particles in the green area}}\times 100.$$

For an ideal trapping behavior induced by pDEP, the number of particles in the red area would be 0 and, therefore, FR = 0%.

For nDEP or case |A|>|B|, the average distance between the electrode edge and particle stream which is guided along the electrodes is measured and averaged over 20 measurement points as shown in Figure 4B. In addition, the width of the focused particle stream at the electrode output is measured and compared.

### 3.2 Trapping Experiments in a Mesh‐Based DEP Filter

An insulator‐based DEP filter with a mesh between two conductively coated glass slides as electrodes is used (Figure 6A). The setup is described in detail in [12]. The coarse mesh woven from polypropylene fibers with a fiber diameter of 340μm acts as a field disturber and generates field maxima on the surface of its fibers. The pore size of the mesh of 500μm is much larger than the particles, so that particles are almost exclusively retained when they are moved and held at the field maxima via pDEP. The applied signal is generated using a Rigol DG4062 signal generator and amplified using a FLC Electronics A400 amplifier. The voltage applied to the channel is measured with a N4L PPA1500 power analyzer (Newtons4th Ltd, UK). 2.48μm polystyrene (PS) particles (FluoGreen, non‐functionalized, MicroParticles GmbH, Germany) are used as model particles. These are dispersed in ultrapure water containing 0.005% Tween20, 6μM potassium hydroxide, and 0.25μM potassium chloride ($\sigma_{m}=1.0\;\mu S\;{\mathrm{cm}}^{-1}$, $2.5\times 10^{5}\;\text{particles per milliliter}$). Particles are pumped at a volume flow of 120 $\mathrm{mL}\;h^{-1}$ through the channel using a piston pump (Ismatec MCP‐CPF IP65 with pump head FMI 202 QP.Q0.SSY, Cole‐Parmer GmbH, Germany). To determine the separation efficiency, the fluorescence intensity is measured at the channel outlet (Figure 6B) using a spectrometer setup (introduced in [12]; EXFO X‐Cite 120 PC Q light source, StellarNet Silver nova spectrometer, Hellma 176‐765‐85‐40 flow‐through cuvette). The initial concentration is recorded for 60 s, then the electric field is applied for 180 s, and the particles are recovered for another 120 s by switching off the field. The separation efficiency is determined between 225 and 245 s using

(22) $$\eta =1-\frac{{\bar{I}}_{\mathrm{DEP}}}{{\bar{I}}_{c0}}$$

for three repetition experiments, with the averaged fluorescence intensity while trapping ${\bar{I}}_{\mathrm{DEP}}$ and the averaged fluorescence intensity of the initial particle concentration ${\bar{I}}_{c0}$. A sinusoidal and rectangular signal with the same measured peak‐to‐peak voltage at the DEP channel of $145\;V_{\mathrm{pp}}$ and a frequency of 15 kHz is used for all experiments.

**FIGURE 5:**
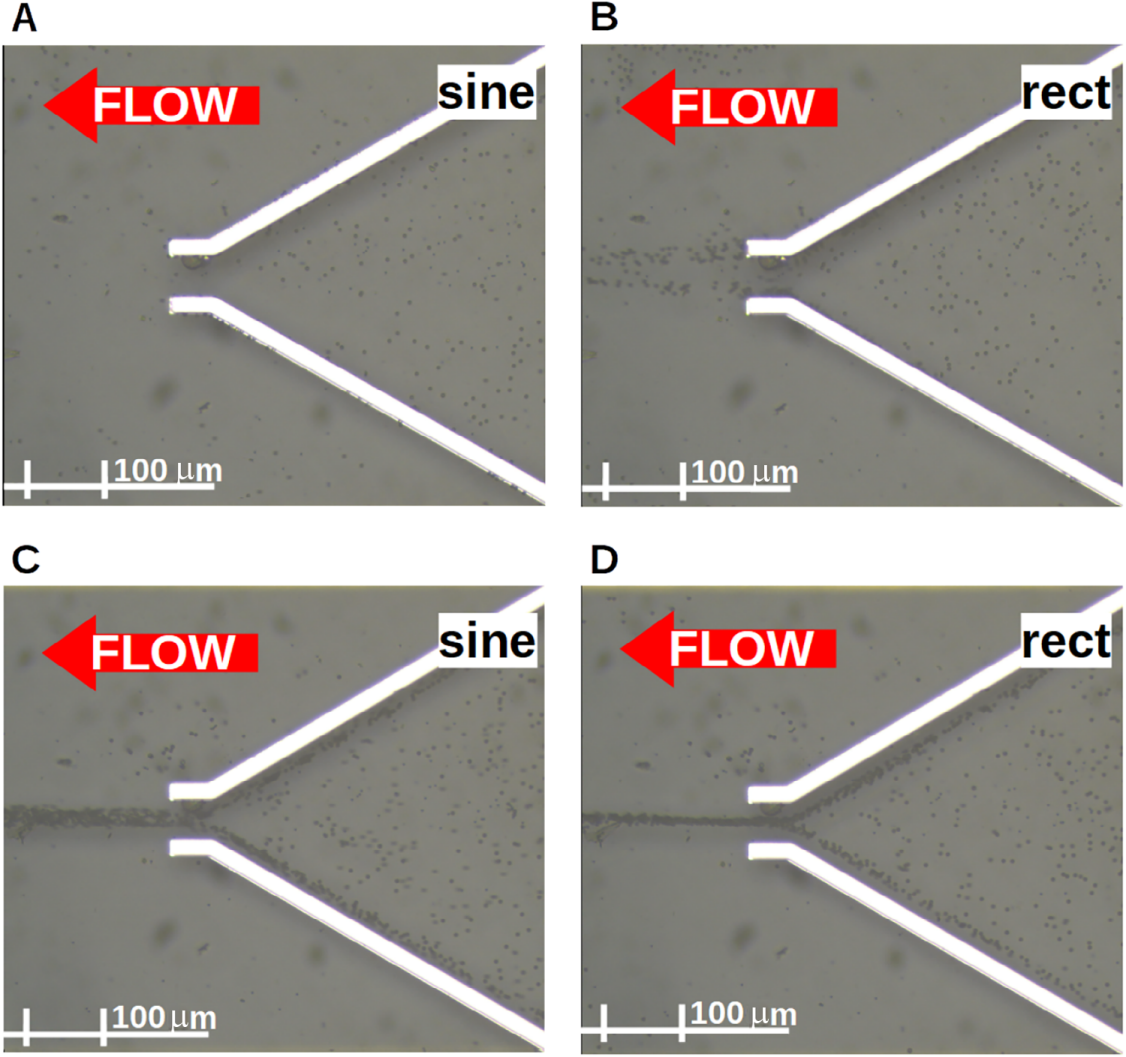
Comparison of the experimental results for sinusoidal (A,C) and rectangular (B,D) signals at an amplitude of $10\;V_{pp}$ and frequencies of 10 kHz and 350 kHz, respectively, using 4.5μm carboxyl functionalized polystyrene microspheres [18] in DI water. The pDEP induced by the rectangular waveform in (B) is diminished by the counter‐orientated force of the harmonics leading to a release of particles, whereas the pDEP induced by the sinusoidal waveform in (A) can trap the particles at the electrodes. The nDEP induced by the rectangular waveform in (D) is increased by the equally orientated forces of the harmonics leading to a higher net force and a stronger focusing of the particles, whereas the nDEP induced by the sinusoidal waveform in (C) has a lower net force and particles are less focused on the electrode structure and the DEP force.

**FIGURE 6:**
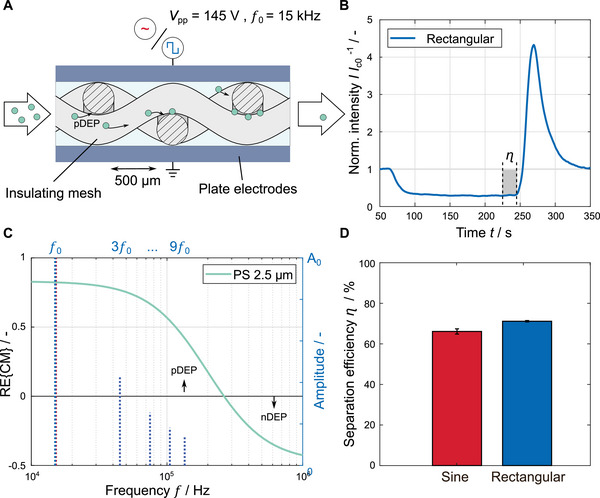
Comparison of the separation efficiency using sinusoidal (red) and rectangular (blue) signals in insulator‐based DEP separation experiments. (A) Principle sketch of the mesh‐based DEP filter (not to scale). The electrically insulating polypropylene mesh creates field maxima on the thread surface, which serve as pDEP trapping spots and retain particles out of the suspension flow. (B) Example of measured fluorescence intensity I normalized to the intensity of the initial particle concentration ${\bar{I}}_{c0}$ at the channel outlet during the separation experiments using a rectangular signal (mean value of three repetitions). A voltage of $V=145\;V_{\mathrm{pp}}$, a frequency of f=15kHz, a volume flow of $120\;\mathrm{mL}\;h^{-1}$, and a medium conductivity of $1.0\;\mu S\;{\mathrm{cm}}^{-1}$ are used. (C) RE{CM(f)} for 2.5μm PS particles. The used frequency and associated harmonic components of the rectangular signal are indicated by dotted lines. (D) Comparison of the separation efficiency between sinusoidal and rectangular signals (three repetitions).

## 4 Results and Discussion

### 4.1 Particle Focusing in an Electrode‐Based Flow Cell Experiment

Since the cross‐over frequency of the CM factor is at approximately 40 kHz, it is expected that for a signal frequency of 10 kHz the harmonic distortions of the rectangular waveform will lead to an overall decrease in the net force. For a sinusoidal waveform, the particles will only experience positive DEP.

Considering Figure 5A for the sinusoidal waveform the particles are attracted towards the electrodes and trapped in between. In Figure 5B, the particles experience a much weaker force induced by a rectangular signal, leading the particles to be pushed along the electrodes to the middle where they are released.

Quantitatively, for the rectangular waveform, the FR is 37.05% and for the sinusoidal waveform it is 16.33%. Although the trapping of the particles with a sinusoidal waveform is much better, a FR of approximately 16% occurs. We assume that this is due to particles coming from the right and are attracted by the pDEP force, having enough energy to push out particles on the other side of the electrodes which are then being dragged away from the electrodes due to the force of the flow.

For $f_{0}=350\;\text{kHz}$, all harmonics lead to an nDEP force which has the same sign as the nDEP force of the fundamental frequency, leading to the assumption that the overall net force will increase in comparison to a sinusoidal waveform.

For Figure 5C, all particles experience a distinct negative DEP force and are repelled from the electrodes using a sine wave. However, as seen in Figure 5D, for a rectangular waveform the particles are repelled much stronger and farther away from the electrodes leading to a much better focus of the particle stream at the outlet of the electrode structure which is beneficial for some applications [9].

The distance between the particle stream and electrode edge for a rectangular waveform is 11.38μm, and the distance for a sinusoidal waveform is 5.05μm. Also, the width of the particle stream at the output can be compared. For a rectangular waveform, it is 9.48μm and for a sinusoidal waveform it is 17.77μm. So the induced force with a rectangular waveform is significantly higher in comparison to a sinusoidal waveform increasing the focusing capability by 93.4%.

We conclude that in comparison to pDEP and nDEP experiments, an increase in DEP force may be observed when using rectangular waveforms, but this does not generally apply. Although effective amplitude and therefore $V_{\text{rms}}$ of the rectangular signal is higher in comparison to the sinusoidal waveforms, the condition of Equation (19) needs to be considered to avoid diminishing effects.

### 4.2 Trapping Experiments in a Mesh‐Based DEP Filter

If a sinusoidal or rectangular‐shaped electrical signal is applied to the mesh‐based channel, the measurable fluorescence intensity at the channel outlet drops significantly (Figure 6B starting at 70 s). For the particle concentration used in the experiments, this intensity correlates directly with the particle concentration passing through the filter. Accordingly, a large number of particles are retained in the channel by DEP. After switching off the electric field, the trapped particles are released and a fluorescence peak occurs (Figure 6B starting at 250 s). Using a sinusoidal signal, a separation efficiency of η=66.1%±1.3% is reached. In comparison, a rectangular signal of the same amplified peak‐to‐peak value achieves a separation efficiency of η=71.2%±0.4% (Figure 6D). For 2.5μm particles (cross‐over frequency of around 260 kHz) and a fundamental frequency of 15 kHz, the relevant first two harmonics of the rectangular signal contribute to the pDEP force (Figure 6C). The condition |A|>|B| in Equation (10) is fulfilled. An average relative improvement of 7.7% is achieved by using the rectangular signal. This is lower than the theoretically expected value as derived from Equation (19). The reason for this could be that the used DEP filter type does not approach a separation efficiency of 100% for certain parameter combinations (mesh size, flow rate, particle load, etc.) despite an increase in the energy input. For the parameter combination used for this study, we observe a saturation effect at around 75%. Bubble formation or a significant temperature increase is not noticeable which could explain the saturation effect. The increase in separation efficiency may therefore not be proportional to the increased energy input due to the harmonics of the rectangular signal. Accordingly, a smaller but still statistically significant increase in separation efficiency can be achieved by switching from a sinusoidal to a rectangular signal.

Besides the investigated differences in DEP force, focusing separation efficiency, no other effects such as electrochemical reactions or others were observed induced by using rectangular waveforms.

## 5 Conclusion

In this article, the influence of the waveform on dielectrophoretic applications was investigated. It was shown that using a rectangular signal, the harmonics of the fundamental frequency can have a significant influence on the separation or sorting efficiency. The harmonics generate an additional DEP force, which can have an amplifying effect if the contribution has the same sign or a diminishing effect for opposite signs. A positive effect could be achieved both for focusing particles showing nDEP and trapping of particles with a cross‐over at a high frequency via pDEP. Furthermore, a showcase for a disadvantageous experiment was shown, where $f_{0}≮\frac{f_{\text{co}}}{5}$. Here, the use of rectangular waveforms resulted in a decrease of the DEP net force leading to the conclusion that the sole consideration of the RMS amplitude is an insufficient metric for deciding if the use is beneficial or not.

Nevertheless, by meeting the aforementioned conditions, changing the waveform from sinusoidal to rectangular may offer a simple approach to improve the efficiency of DEP processes without altering the experimental setup (see Supporting Information).

## Author Contributions

N.P.B. and L.W. conceptualized the experiments, N.P.B., J.M.S., and L.W. conducted the experiments, and all authors analyzed and discussed the results and reviewed the manuscript.

## Conflicts of Interest

The authors declare no conflicts of interest.

## Supporting information

Supporting Information

## Data Availability

Data are openly available in a public repository that issues datasets with DOIs [22].
