# Supplementary material for: When to Use Rectangular Waveforms in Dielectrophoresis Application to Increase Separation and Sorting Efficiency
Source: Electrophoresis. 2024 Nov 28;46(1-2):104–11. doi: 10.1002/elps.202400164 (PMC11773296; doi:10.1002/elps.202400164)
Supplement: Supplementary file 1 — Supporting Information [file ELPS-46--s001.pdf]

When to Use Rectangular Waveforms in Dielectrophoresis Application to Increase Separation and Sorting Efficiency

Niklas P. Boldt, Laura Weirauch, Jana M. Späth, Uwe Kerst, Mario Birkholz, Michael Baune, Roland Thewes

Supporting Information

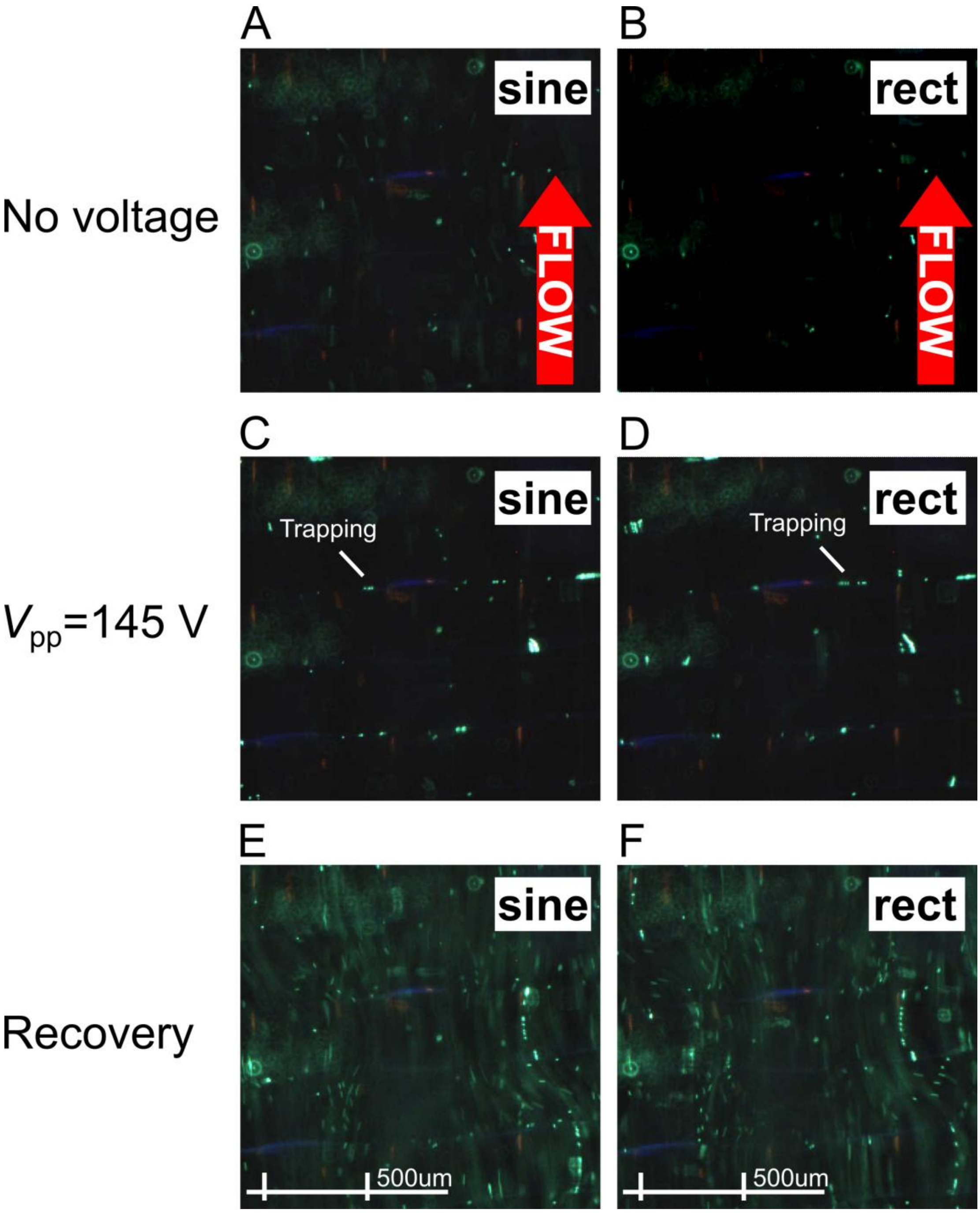

Figure S1: Comparison of a sinusoidal **(A,C,E)** and rectangular **(B,D,F)** signal applied to a mesh-based DEP filter. **(A,B)** show the particle flow at 120 mL h^{-1} without applied voltage. In **(C,D)** a voltage of V = 145 V_{pp} and a frequency of f = 15 kHz are used to trap particles in the mesh. In **(E,F)** the particles are released by switching off the electric field.
